# Liquid tumor microenvironment enhances *WNT* signaling pathway of peritoneal metastasis of gastric cancer

**DOI:** 10.1038/s41598-023-38373-6

**Published:** 2023-07-10

**Authors:** Huawei Xu, Zhibin Hao, Yujie Wang, Deng Zhang, Jie Li, Ling Chen, Ninghua Yao, Binbin Qian, Xiaobo Peng, Xianbao Zhan

**Affiliations:** 1 Department of Oncology, Changhai Hospital, Naval Military Medical University, Shanghai, 200433 China; 2 Department of Oncology, Tongzhou People’s Hospital, Nantong, 226300 China; 3 Research and Early Development, Haobai Biotechnology Inc, Shanghai, 200235 China; 4 Department of Oncology, Affiliated Hospital of Nantong University, Nantong, 226000 China

**Keywords:** Metastasis, Gastric cancer, Cancer models

## Abstract

Gastric cancer remains one of the most prevalent tumors worldwide and peritoneal metastasis is responsible for approximately 60% of death in advanced gastric cancer patients. However, the underlying mechanism of peritoneal metastasis is poorly understood. We have established organoids derived from malignant ascites (MA) of gastric cancer patients and noticed that MA supernatant could strongly increase the colony formation of organoids. Thus, we realized the interaction between exfoliated cancer cells (ECCs) and liquid tumor microenvironment contributes to peritoneal metastasis. Further, we designed a medium component control test which proved that exosomes derived from MA could not enhance the growth of organoids. Using Immunofluorescence and confocal imaging as well as dual-luciferase reporter assay, our data showed *WNT* signaling pathway was upregulated by high concentrations of *WNT* ligands (wnt3a and wnt5a), which was verified by ELISA. Besides, suppressing *WNT* signaling pathway diminished the growth promoting function of MA supernatant. This result implicated *WNT* signaling pathway as a potential therapeutic target for peritoneal metastasis of gastric cancer.

## Introduction

Metastasis is the major driver of death in patients with cancer. Particularly peritoneal metastasis is responsible for approximately 60% of death in advanced gastric cancer patients^1^. Malignant ascites (MA), as one of the most common symptoms of advanced gastric cancer patients, predicts adverse prognosis.

The occurrence of implantation metastasis involves the dissemination of ECCs and pre-metastatic niche formation. Mesothelial-mesenchymal transition (MMT) is considered the key to a series of pathological changes^2^.

MA is a hypoxic inflammatory and immunosuppressive tumor microenvironment, composed of complex cytokine chemokine, growth factors, exosomes, and various suspension cells such as tumor mesothelial cells and immune cells. However, the interaction between ECCs and tumor microenvironment, such as the avoidance of anoikis, is still poorly understood.

Up to now, organoid lines derived from various kinds of tumors have been established and proved high consistency in biological inheritance^3^. A mass of research meaning to verify the feasibility, that organoids are employed for individualized drug screening, hosted on one after another around the beginning of the pilot^4–6^. The significance of the tumor microenvironment in tumor genesis and progression is becoming recognized as research progresses. Tumor stroma including immune cells has been brought to the organoid culture system to mimic the tumor microenvironment^7,8^.

Organoids modeling normal gastric units and gastric cancer have been reported before^9^. Using a similar protocol, we established organoids derived from MA of gastric cancer patients and we noticed that MA supernatant could strongly increase colony formation of malignant ascites-derived organoids (MADO)^10^. We considered the heterogeneity both between primary gastric cancer and ECCs, as well as their discrepant microenvironments, caused this phenomenon occurs. Actually, the diversity of the mutation spectrum between primary tumors and ECCs in MA has been reported before^11^.

Therefore, we set out to determine how the MA microenvironment enhances the malignant biological behaviors of ECCs. Here we show that there are high levels of *WNT* ligands (wnt3a and wnt5a) in ascites supernatant, which upregulated *WNT* signaling pathway.

## Materials and methods

### Human specimens

MA was collected from gastric cancer patients undergoing palliative paracentesis. All these cases were pathologically confirmed as gastric adenocarcinoma by surgery or endoscopic biopsy, and their ascites were confirmed to contain tumor cells by liquid-based thin-layer cytology. All patients involved signed an informed consent. All tissue collections and experiments were conducted in strict accordance with the guidelines of the Declaration of Helsinki (2013) and were approved by the local ethics committee (Committee on Ethics of Medicine, Navy Medical University, PLA). The specific ethics documents can be found electronically in the Related Files section of this article.

### Tumor cells isolation and culture

Abdominal puncture drainage was performed clinically, and the ascites in the drainage bag were collected into a dry sterile bottle of about 200 ml. All these samples were transported to the laboratory at the temperature of 4 °C, while experimental treatment was carried out within 6 h.

Upon arrival, MA was filtered with a 70 μm membrane in order to remove solid suspensions. MA was then centrifuged at 1200 rpm for 5 min in order to enrich EECs. The supernatant was incubated at 56 °C for 30 min and filtered with 0.22 μm membrane after being centrifuged at 3000 rpm for 10 min. Meanwhile, enriched EECs were collected, and seeded into growth factor-reduced Matrigel (Corning 356,231) in a prewarmed 24-well cell culture plate (Costar, 3524) with 400 cell clusters or 1000–2000 single cells in a well. 500 μl of MADO culture medium (Supplementary Table 1) or the mixture of supernatant above and MADO culture medium was added to each well and plates transferred to 37 °C /5% CO_2_ incubators.

The medium was renewed every three days. We make continuous observations of MADOs and take pictures when appropriate. Passaging of MADOs was performed essentially as described by Jie Li et al.^10^. Specifically, MADOs were removed from the Matrigel, mechanically dissociated into small fragments, and transferred to fresh Matrigel with fresh culture medium as described above.

### Cell proliferation/growth assays

Cell proliferation/growth was assessed by CCK8 assays (HY-K0301, MCE) following the manufacturer’s instructions. Briefly, cells were seeded in triplicate in 96-well plates at a density of 100 cell clusters/10 μl Matrigel as described above. Dye solution (DMEM/F12: CCK-8 kit = 10:1) was added at the indicated time points(Day 1, Day 3, Day 6, and Day 9), and the plates were incubated at 37 °C for 1 h before the absorbance was detected at 450 nm. Each test was conducted in triplicate. The culture media were subsequently refreshed.

### Western blot (WB)

For WB, cells were collected and lysed in RIPA buffer (Cell Signaling) with a complete protease inhibitor cocktail (Roche), phosphatase inhibitors (Roche), and PMSF (Sigma), then centrifuged at 12,000*g* for 10 min at 4 °C. Protein concentration was measured by Bio-Rad protein assay kit (Hercules, CA) according to the manufacturer’s instructions. Western blot assays were performed as previously described^12^. The primary antibodies were obtained as follows: anti-caspase-3, anti-PCNA, anti-β-Catenin, anti- Non-phospho (Active) β-Catenin, and Exosomal Marker Antibody Sampler Kit antibody (Flotillin-1 and EpCAM) from Cell Signaling Technology, anti-β-actin antibody from Abmart.

### Preparation of exosomes

The supernatant collected above was centrifuged at 20,000×*g* for 30 min at 4 °C to remove detached cells. The supernatant was then centrifuged at 150,000×*g* for 2 h at 4 °C to remove cell debris. The transparent gelatinous precipitate was resuspended in cold PBS and then the last step above was repeated for further refinement. For protein assay, 10 × RIPA buffer containing 10% PMSF and phosphatase inhibitors was added to the exosome fraction, incubated on ice for 15 min, and applied to the micro BCA protein assay system (ThermoFisher Scientific).

### Transmission electron microscopy (TEM)

The exosome samples were negatively stained as previously described^13^. A 400-mesh copper grid coated with formvar/carbon films was hydrophilically treated. The exosome suspension (5 to 10 μl) was placed on Parafilm, and the grid was floated on the exosome liquid and left for 15 min. The sample was negatively stained with 2% uranyl acetate solution for 2 min. Exosomes on the grid were visualized with 40,000 times magnification with an H-7650 transmission electron microscope (Hitachi, Tokyo, Japan) at Central Research Laboratory, Second Military Medical University.

### Immunofluorescence and confocal imaging

MADOs were cultured on Confocal 35-mm Coverglass-Bottom Petri-Dishes (BIO-IMAG). Immunofluorescence and confocal imaging were performed as previously described^14^. After growing to a suitable density, cells were fixed with 4% paraformaldehyde, permeabilized in 0.5% Triton X-100, blocked with PBS containing 10% goat serum, and incubated overnight with antibodies at 4 °C. Fixed and stained dishes were washed 3 times with PBS, and primary antibodies were labeled by incubation with Alexa Fluor-conjugated secondary antibodies (Invitrogen). The dishes then were mounted with a mounting medium containing DAPI (Prolong Gold Antifade Reagent, Life Technologies), and cells were visualized with confocal laser microscopy (Carl Zeiss).

### Dual-luciferase reporter assay

Dual-luciferase reporter assay was performed as previously described^15^. Specifically, MGC-803 cells were plated in 24-well plates containing PDO media and MA media. Then cells were cotransfected with TOP plasmids after being cultured for three days. Cells were collected 24 h after transfection, and luciferase activities were analyzed by the dual-luciferase reporter assay kit (Beyotime, RG027). Reporter activity was normalized to the control Renilla.

### *WNT* signaling inhibition assay and porcupine invalidation assay

After MADOs were subcultured and tumor cells were expanded to a sufficient number, the mechanically separated MADOs fragments were evenly mixed with matrigel (Corning 356231) and seeded into 96-well cell culture plate (Costar, 3516) with 100 cell clusters or 250–500 single cells per well. The drug sensitivity assay was conducted when every well contained about 200 ± 50 MADOs in 15 mL matrigel with 300 mL culture medium. Salinomycin (MCE, HY-15579) and porcupine inhibitor (Porcn-IN-1, MCE, HY-111472) were prepared in MADOs medium according to the different concentrations (tenfold dilution). After MADOs were grown to suitable conditions, the prepared medium with drug gradient concentrations was added. After 3 days of drug treatment, the drug-containing medium was refreshed and another 3 days of drug treatment was repeated. Then the cell viability of MADOs in each well was detected. According to the manufacturer's instructions, MADOs were treated with Cell Counting Kit-8 (MCE, HY-K0301), and the OD value of each well was detected by Cytation5 (BioTek). The OD (optical density) values of the dosing group and the control group were converted to the survival rate of MADOs in each well, and then Graphpad Prism8 was used to form a fitting curve between different Salinomycin concentration (lg) and the survival rate of MADOs. The half maximal inhibitory concentration (IC50) of Salinomycin in MADOs was calculated, and an appropriate drug concentration was selected for the *WNT* signaling pathway inhibition experiment. Each test was conducted in triplicate. The concentration of Porcn-IN-1 is referred to the MCE guidelines (https://www.medchemexpress.cn/Porcupine-IN-1.html), 0.5 ± 0.2 nM, and we finally choose the appropriate drug concentration according to the actual situation.

### ELISA

During the aforementioned MADOs culture, the medium supernatant was collected every 3 days. The samples were centrifuged at 1000 rpm for 3 min at 4 °C to eliminate any potential cellular components, and the medium was separated and stored at − 80 °C within 2 h for further analysis. Wnt-3a/Wnt-5a levels in medium samples were measured using commercially available enzyme-linked immunosorbent assay (ELISA) kits, according to the recommendations of the manufacturer (CUSABIO, CSB-EL026136HU/CSB-EL026138HU, Houston, USA). All measurements were performed in double for each sample, and the mean value was calculated.

### Statistical analysis

The average fluorescence intensity of MADOs were measured using ImageJ as previously described^16^. Statistical analyses were performed using Prism GraphPad. One-way ANOVA was used for multi-group comparisons. All values are shown as mean ± standard error of the mean (s.e.m.). P ≤ 0.05 was considered statistically significant.

### Ethics statement

This study was approved by the Ethics Committee of Changhai Hospital of Second Military Medical University (CHEC2016-157), and written informed consent was obtained from all patients or their guardians.

## Results

### MA supernatant enhances the growth of MADOs

**Figure 1:**
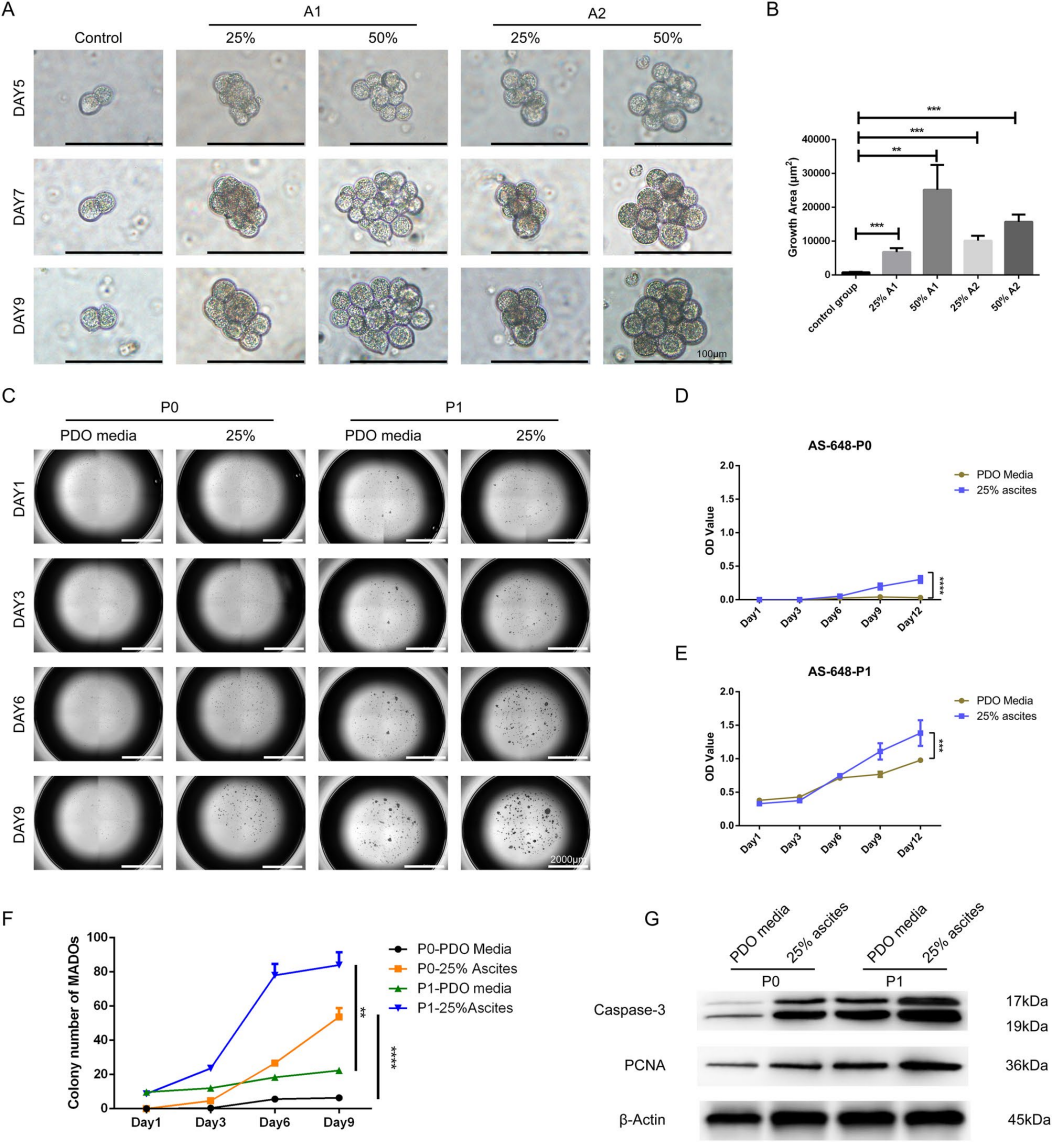
Both parental and non-parental supernatant of ascites promote growth of MADOs. (**A**) Representative bright-field images of one organoid line, which was derived from a patient (P1) with gastric carcinoma, indicating that growth of MADOs was increased by adding indicated proportion of supernatant derived from parental ascites (A1:25%, 50%) or another patient’s (P2) counterpart (A2:25%, 50%). Scale bar, 100 μm. (**B**) Histogram presenting growth area of MADOs treated above (Day 9). Data indicated mean ± S.D. and analysed by Student’s *t* test (**p < 0.01; ***p < 0.001). (**C**) Representative images of primary culture (P0) and subculture (P1) of MADOs taken under Agilent BioTek Cytation, treated with PDO media or media containing 25% ascites supernatant. Scale bar, 2000 μm. (**D**,**E**) Proliferation of P0 and P1 MADOs as determined by CCK8 assays. (**F**) Colony number counting of P0 and P1 MADOs above. (**G**) Western blotting analysis of Caspase-3 and PCNA in MADOs above (Day 9).

Using time-lapse photography, we could describe changes in the single organoid. MA supernatant significantly increased the forming efficiency and size of organoids by promoting cell division. To rule out whether the individual difference come from tumor cells or tumor microenvironment, we designed a cross-matching test that MADO lines were incubated with parentally or non-parental supernatant of ascites respectively (Fig. 1A,B, Supplementary Fig. 1). As expected, both parental and non-parental supernatant of ascites increased the colony formation of MADOs. We wonder if MA supernatant has the same effect on subcultured MADOs. Not surprisingly, we observed increased cell proliferation and colony formation both in primary MADOs (P0) and subcultured MADOs (P1) by CCK8 assays colony number counting (Fig. 1C–F). Besides, primary tumor cells hardly form organoids without MA supernatant, while MA supernatant seems to only affect the growth rate of P1 MADOs. Interestingly, we noticed that both proliferation and apoptosis of MADOs were increased when they were stimulated by MA supernatant (Fig. 1G). We, therefore, hypothesized that there is a common substance contributing to the growth promotion effects on MADOs in ascites.

### MA supernatant-derived exosomes cannot enhance the growth of MADOs

Besides various suspended cells like mesothelial cells and immune cells, complex cytokines, chemokines, growth factors, and exosomes constitute the extracellular environment of EECs. To determine whether exosomes directly enhance the growth of MADOs, we purified exosomes from MA by the ultracentrifugation method drawing on and improving the method reported by Yanting Hu^13^. Transmission electron microscopy showed the size distribution of purified exosomes was between 40 and 100 nm with classical discal morphology (Supplementary Fig. 2). Moreover, Western blot analysis of protein extracted from exosomes revealed the presence of specific exosome markers (Flotillin-1 and EpCAM). Taken together, these results indicated that the main contents of the purified microvesicles were exosomes.

**Figure 2:**
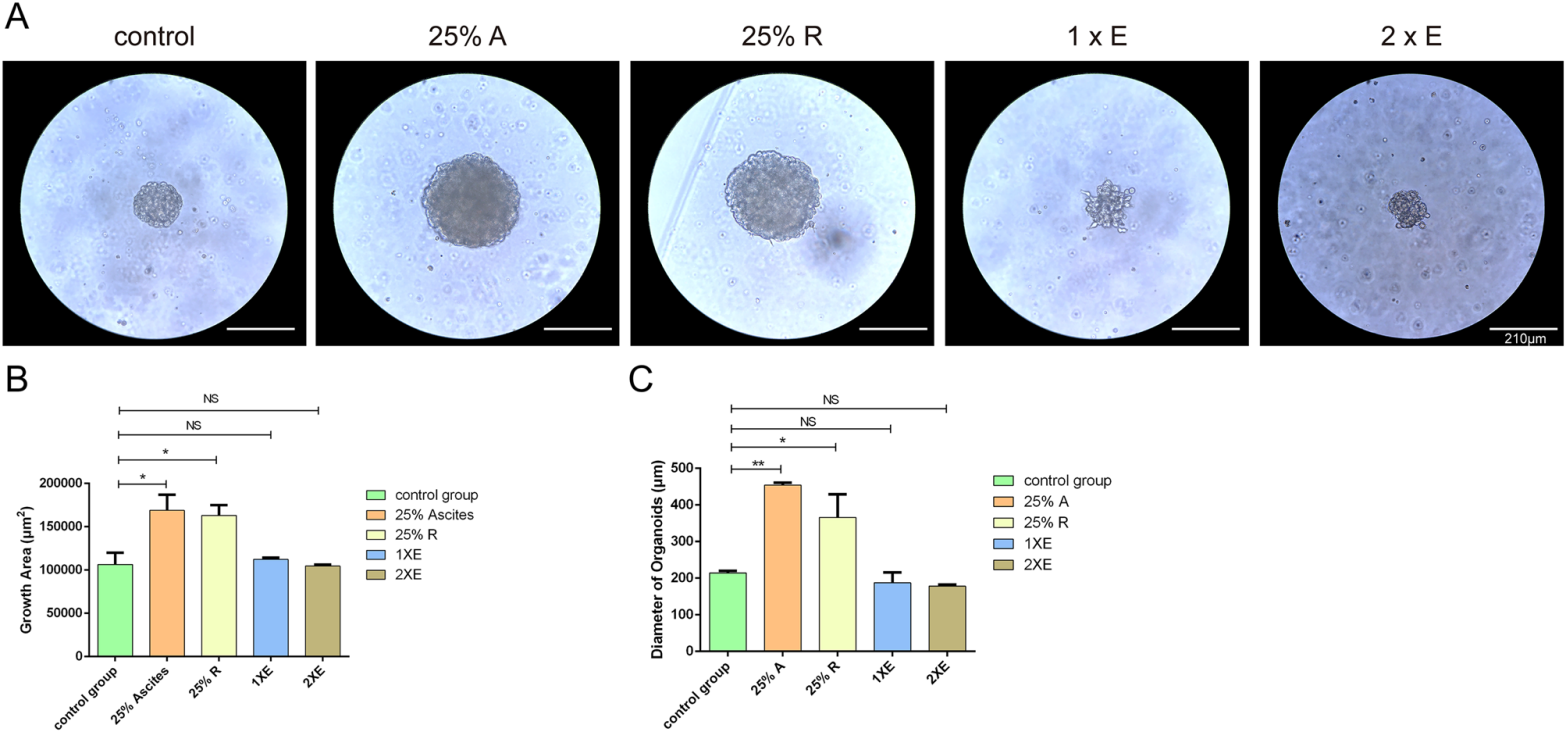
Exosome derived from ascites cannot promote growth of MADOs. (**A**) Representative bright-field images of one MADOs treated by supernatant of ascites (A), exosome isolated from the same and double volume of the ascites (1xE, 2xE), and the remaining part of the ascites (R). Scale bar, 210 μm. (**B**) Histogram presenting growth area of MADOs treated above. (**C**) Histogram presenting diameters of single MADO treated above. Data indicated mean ± S.D. and analysed by Student’s t test (*p < 0.05; **p < 0.01; NS p > 0.05).

To rule out the deviation probably caused by the biological dysfunction of exosomes during the extraction process, we performed a media component control test that MADOs were stimulated by exosomes, parental MA supernatant (25% here), and the remaining part of the MA supernatant. We observed that exosomes had no significant effect on the growth of MADOs. Conversely, the remaining part group strongly promoted the growth of MADOs, which was very close to the MA group (Fig. 2). This test demonstrated exosomes cannot directly enhance the growth of MADOs, that is to say, the substances contributing to the growth promotion effects on MADOs in ascites do exist in the supernatant of MA instead of exosomes.

### *WNT* signalling pathway is activated under the stimulation of MA supernatant

To figure out the growth-promoting effect produced through which signalling pathway, we used western blotting assays to test key protein-expressing levels of several gastric cancer cell lines (Supplementary Fig. 4). We detected elevated levels of Non-phospho β-catenin, also known as active β-catenin in the *WNT* signaling pathway, after being stimulated by MA supernatant (Supplementary Fig. 3). Meanwhile, the level of total β-catenin expression stayed the same after treatment. This result suggested the canonical *WNT* signaling of gastric cancer cell lines is upregulated when they were stimulated by MA supernatant.

**Figure 3:**
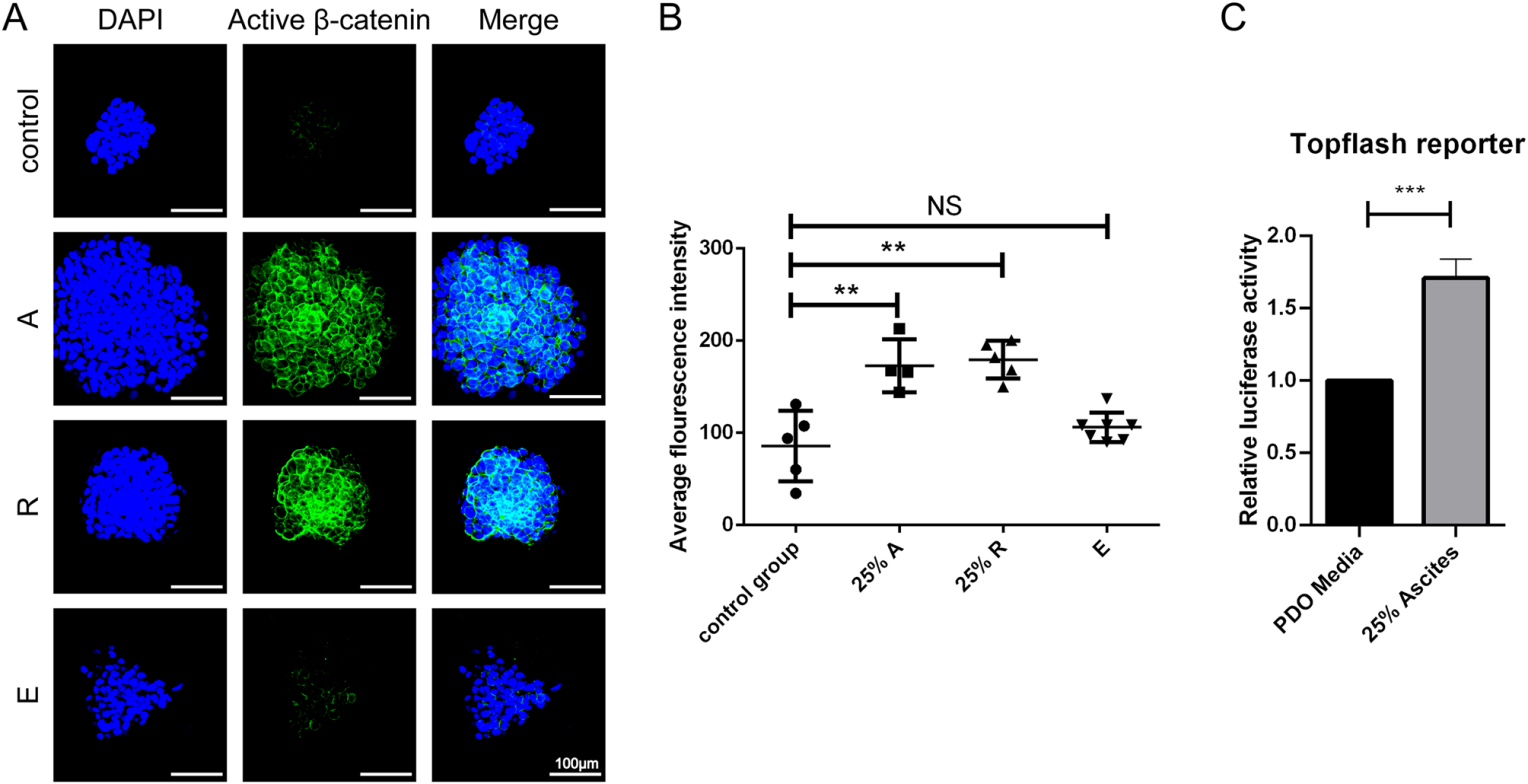
Supernatant of ascites upregulated signaling of active β-catenin. (**A**) Immunofluorescence for the localization of active β-catenin in MADOs treated by supernatant of ascites (A), exosome isolated from the same volume of the ascites (E) and the remaining part of the ascites (R). Scale bar, 100 μm. (**B**) Statistical scatter plots presenting average fluorescence intensity of MADOs treated above. Data indicated mean ± S.D. and analysed by Student’s *t* test (**p < 0.01; NS p > 0.05). (**C**) Histogram presenting relative luciferase activity of Topflash reporter in AGS-803 cells. Data indicated mean ± S.D. and analysed by Student’s *t* test (*p < 0.05; **p < 0.01; ***p < 0.001; NS p > 0.05).

To further address the same effect on MADOs, we detected the expression level of non-phospho β-catenin before and after treatment. We employed a semiquantitative method to analyze the intensity of the fluorescence signal (Fig. 3). As expected, both the MA group (M) and the remaining part group (R) showed a higher fluorescence signal after treatment, except the exosome group (E).

After being stimulated by MA, the activation of Wnt/β-catenin signaling in MGC-803 was further evidenced by the high luciferase activity of the topflash reporter (Fig. 3C). To sum up, these results indicated that the *WNT* signaling pathway in MADOs is activated after being stimulated by MA supernatant.

### High levels of wnt3a and wnt5a exist in MA supernatant

**Figure 4:**
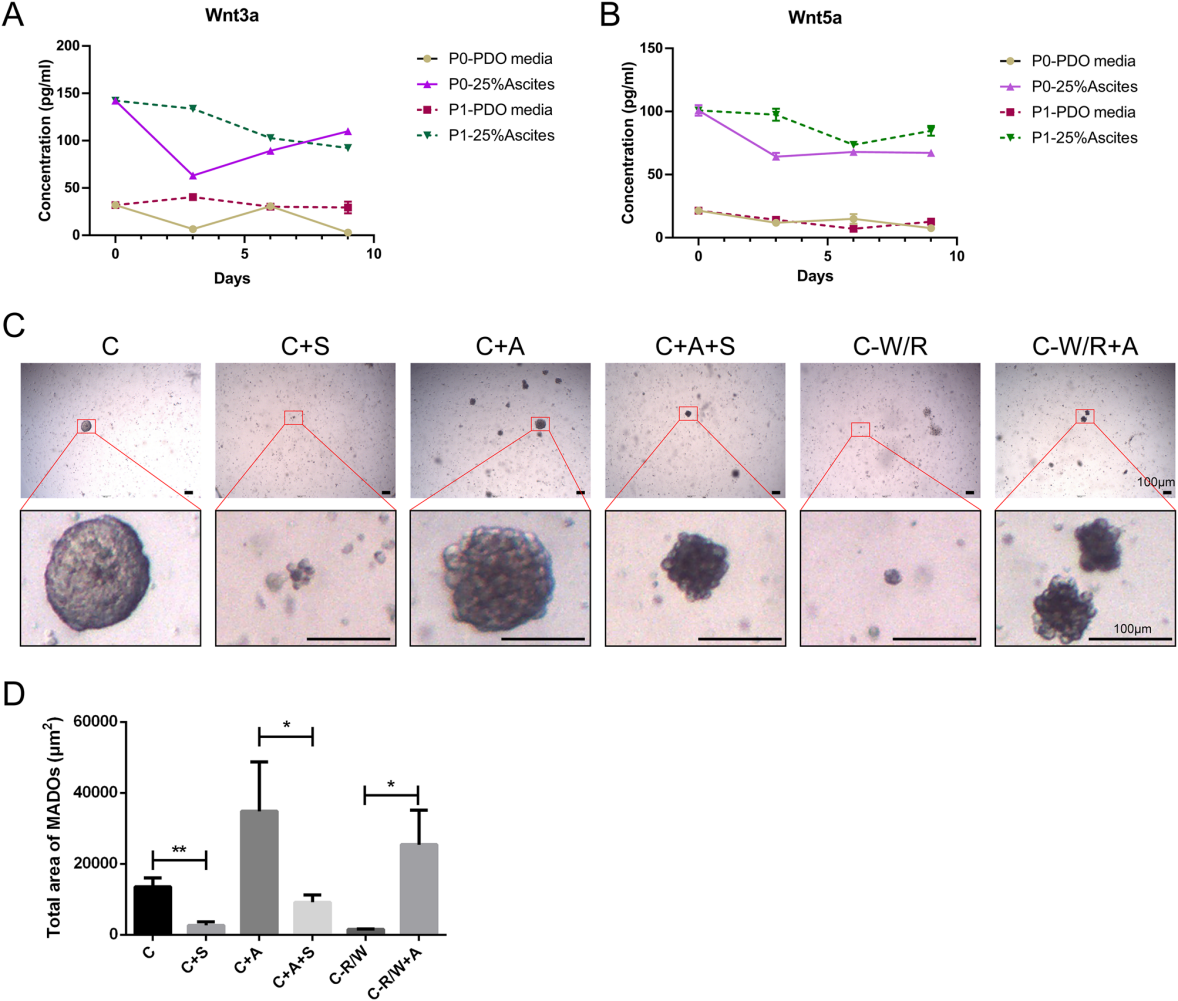
High concentration of wnts in ascites promote growth of MADOs, which could be suppressed by blocking *WNT* signaling. (**A**,**B**) Line charts indicate the concentration of wnts in culture media during P0 and P1 MADO culture at indicated time points (Day 0, Day 3, Day 6 and Day 9), as measured with ELISA. Statistical analysis proves significant differences between PDO media group and 25% ascites group at every testing point. (**C**) Representative bright-field images of one MADOs treated by Salinomycin (S), supernatant of ascites (A), removing Wnt 3a/R-spondin 1 (− W/R). (**D**) Histogram presenting growth area of MADOs treated above. Data indicated mean ± S.D. and analysed by Student’s t test (*p < 0.05; **p < 0.01).

We detected the concentration of wnt3a and wnt5a both in PDO media and media containing 25% MA supernatant at indicated time points (Figs. 1C, 4A,B, Table S2). We found the level of wnt3a and wnt5a in MA supernatant were approximately 13 and 15.8 times than PDO media. Interestingly, wnt3a/wnt5a in culture media decreased at first and then increased during the culturing of MADOs, which indicated a dynamic level of wnt3a/wnt5a during the culture of MADOs.

### MADOs autocrine WNT ligands can be inhibited by Porcupine inhibitor

When MADOs were cultured for 12 days, the concentration of Wnt3a and Wnt5a in culture media even overpassed the original counterpart (Supplementary Fig. 5A). This is compelling evidence that autocrine exists during the culture of MADOs. Then we used Porcupine inhibitor (Porcn-IN-1) to inhibit the secretion of Wnts at day 9, when was the inflection point of concentration changes. Despite no statistical significance, there was a downtrend in Wnt3a and Wnt5a when Porcn-IN-1 was at the concentration of 5 nM (Supplementary Fig. 5C,D). However, we noticed there was a significant decline in the proliferation (Supplementary Fig. 5B).

### The function of MA supernatant can be weaken by suppressing WNT signal

To confirm whether the activation of the *WNT* signal leads to the accelerating growth of MADOs, we performed a *WNT* signal inhibition assay to observe the growth of MADOs. Here we utilized Salinomycin, which blocks Wnt-induced LRP6 phosphorylation, to inhibit Wnt/β-catenin signaling. In the first place, we performed a drug sensitivity assay to confirm suitable drug concentration to MADOs. Referring to the previous report, we set up six gradient concentrations (100 uM, 10 uM, 1 uM, 0.1 uM, and 0.01 uM) to test the response of four candidate MADOs^17^. Then the reproducible dose–response curves and the half-maximal inhibitory concentrations (IC50) were generated and identified (Supplementary Fig. 6). The IC50 values range from 0.024 to 0.1971 uM and the maximal value (0.2 uM here) was chosen to test the response of MADOs.

As expected, the growth of MADOs was inhibited whether treated by *WNT* inhibitor Salinomycin or cultured under *Wnt* withdrawal. We noticed that the accelerating effect contributed by MA supernatant was eliminated when the *WNT* signal was blocked. However, the growth inhibition caused by *Wnt* deletion was reversed by MA supernatant (Fig. 4C,D). Taken together, these data indicated that MA supernatant strongly promotes the growth of MADOs via activating the *WNT* signaling pathway.

## Discussion

The phenomenon we found during optimizing the MADO culture condition suggests the current artificial niche is insufficient to simulate the microenvironment of EECs. The first thing we need to figure out is whether the growth-promoting effect is specific or not, scilicet if the effect exists only between EECs and parental ascites supernatant. Through the cross-matching test, we found both parental and non-parental supernatant of ascites promoted the growth of MADOs. Moreover, MA supernatant could strongly promote the growth of subcultured MADOs. Taken together, these data indicated the growth promotion of MA supernatant on MADOs is universal. We noticed that primary tumor cells were more dependent on MA supernatant than subcultured MADOs. This may be associated with differences between primary tumor cells and organoid structures.

Besides complex cytokines cocktail, abundant exosomes suspend in ascites. In view of the mature technology extracting exosomes, we first tested the exosome to verify if it is the initiating agent. The result showed exosomes cannot directly enhance the growth of MADOs. Interestingly, we noticed that exosomes lead to a morphological change of MADOs turning from spherical to loose morphology. Yanting Hu et al. demonstrated that malignant ascites-derived exosomes from GC patients promote peritoneal tumor cell dissemination via promoting EMT signaling^13^. These data indicated exosomes in ascites mainly promote peritoneal tumor cell dissemination rather than proliferation.

Different from para-carcinoma normal tissue to tumor tissue, MA lacks corresponding contrast. Previous studies attempted to find protein markers by comparing MA with other kinds of ascites, such as cirrhosis ascites^18^. This method is unsuitable for difference selection on account of the huge error caused by individual differences.

Hence, we set about investigating from downstream to elucidate the underlying mechanism. In view of long period and high cost of conducting western blot analysis on organoids, we used several cell lines (SNU5, SNU16, AGS, MKN28 and MGC803) to test a series of key proteins of several classical signaling pathway related to proliferation such as β-catenin, mTor, p-Stat3, p-ERK. The results showed canonical *Wnt*/β-Catenin signaling was activated after being stimulated by ascites supernatant. We further demonstrated that MA supernatant upregulated the *WNT* signaling pathway of MADOs. ELISA validated higher levels of *WNT* ligands (wnt3a and wnt5a) in MA supernatant than in PDO media.

It has been widely discussed recently that Wnts activate the WNT signalling pathway by autocrine^19,20^. Here we demonstrated autocrine WNT ligands exist during the culture of MADOs. This could, at least partly, contribute to the high concentration of WNT ligands in MA. We also noticed the downtrend of Wnts concentrations and growth inhibition effects when the Porcupine inhibitor was used. This illustrated the important role of wnt ligands in MA from the perspective of cell growth statuses. It is also worth mentioning that the effect of blocking Porcupine might be underestimated in vitro because the MA supernatant provided was fixed.

Considering both the growth situation and changes in the *WNT* ligands concentration, we suppose two different stages during the MADO culturing. In the early stage, tumor cells rely on high concentrations of *WNT* ligands to “launch” and *WNT* ligands are consumed strongly during this period. In the late stage, autocrine of *WNT* ligands seize advantage as sphere-forming and tumor cells increased, while high levels of *WNT* ligands initiate positive feedback to promote the growth of MADOs. Interestingly, it seems that primary tumor cells rely on higher levels of *WNT*s than organoids. This may be correlated with improved resistance to low *WNT* ligands environment of MADOs by sphere-forming and evolution.

The *WNT* signal transduction cascade is conserved in evolution and is related to myriad biological phenomena. How it controls stem cells and contributes to diseases has been described by Roel Nusse et al.^21^. *Wnt* pathway agonist R-spondin, epidermal growth factor EGF, and the BMP inhibitor Noggin constitute an artificial niche for organoid in vitro self-renewing. Seidlitz T, et al. observed the dependency level of the *Wnt* signal varies greatly in GC organoids derived from different patients^22^. Nanki et al. uncovered mechanisms that GC organoids acquire *Wnt*-independent phenotype, namely the self-secretion of *Wnt*, APC mutations, and epigenetic WRi regulation^23^. Here, our study supplemented the important role of *WNT* signaling in gastric peritoneal metastasis.

Here, our study lacks detection of APC mutation which can result in self-activation of the *WNT* signaling pathway seems to explain the divergent responses of MADOs to MA supernatant.

## Conclusion

In this study, we made a preliminary inquiry into the mechanism that ascites supernatant increase colony formation of MADOs. We found exosomes cannot directly enhance the growth of MADOs. Hereby, we demonstrated that high concentrations of *WNT* ligands in malignant ascites upregulate the activation of *Wnt*/β-Catenin signaling pathway and we further showed the feasibility of targeting *WNT* signaling pathway.

## Supplementary Information

Supplementary Legends.

Supplementary Figure S1.

Supplementary Figure S2.

Supplementary Figure S3.

Supplementary Figure S4.

Supplementary Figure S5.

Supplementary Figure S6.

Supplementary Information.

Supplementary Table S1.

Supplementary Table S2.

## Data Availability

The original contributions presented in the study are included in the article/supplementary material. Further inquiries can be directed to the corresponding authors.
