# Supplementary material for: Liquid tumor microenvironment enhances WNT signaling pathway of peritoneal metastasis of gastric cancer
Source: Sci Rep. 2023 Jul 10;13:11125. doi: 10.1038/s41598-023-38373-6 (PMC10333202; doi:10.1038/s41598-023-38373-6)
Supplement: Supplementary file 1 — Supplementary Legends. [file 41598_2023_38373_MOESM1_ESM.docx]

**ADDITIONAL FILES**

**Additional file 1: Figure S1.** The growth of an organoid line derived from another patient (P2) with gastric carcinoma was accelerated by adding indicated proportion of supernatant derived from parental supernatant of ascites (A2) or non-parental counterpart (A1). Scale bar, 100 μm.

**Additional file 2: Figure S2.** Characterization of exosomes derived from malignant ascites. (**A**). TEM images of exosomes derived from gastric malignant ascites. (**B**). Western blot detection of proteins in ascites-derived exosomes using antibodies against exosomal markers: Flotillin-1, EpCAM. These exosomes derived from three gastric cancer patients (P1, P2, P3).

**Additional file 3: Figure S3.** (A). Supernatant of ascites promoted growth and aggregation of gastric cancer cell lines (SNU-5, SNU16). (**B**) Western blotting analysis of total β-catenin, active β-catenin and β-actin.

**Additional file 4: Figure S4.** Western blotting analysis of key proteins of several classical signaling pathway related to proliferation in several gastric cancer cell lines stimulated by ascites.

**Additional file 5: Figure S5.** Line charts indicate the concentration of Wnt3a and Wnt5a in culture media during ascites-stimulated MADO culture at indicated time points (Day0, Day3, Day 6, Day 9 and Day 12), as measured with ELISA.

**Additional file 6: Figure S6.** Dose response curves and half-maximal inhibitory concentrations (IC50) of 4 candidate MADOs treated with Salinomycin.

**Additional file 7: Table S1.** MADO culture media

**Additional file 8:** **Table S2.** ELISA detected concentrations of wnt3a/wnt5a in PDO media and media containing 25% MA supernatant.
