# Supplementary figures and images for: Liquid tumor microenvironment enhances WNT signaling pathway of peritoneal metastasis of gastric cancer
Source: Sci Rep. 2023 Jul 10;13:11125. doi: 10.1038/s41598-023-38373-6 (PMC10333202; doi:10.1038/s41598-023-38373-6)

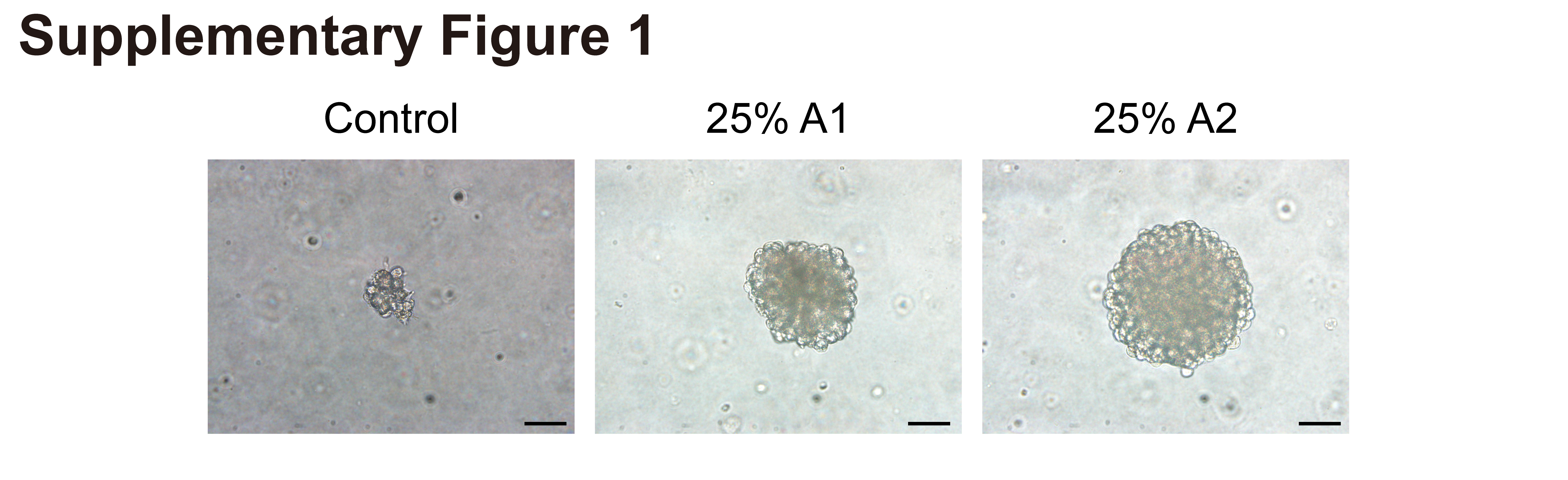

Supplement: Supplementary file 2 — Supplementary Figure S1. [file 41598_2023_38373_MOESM2_ESM.tif]

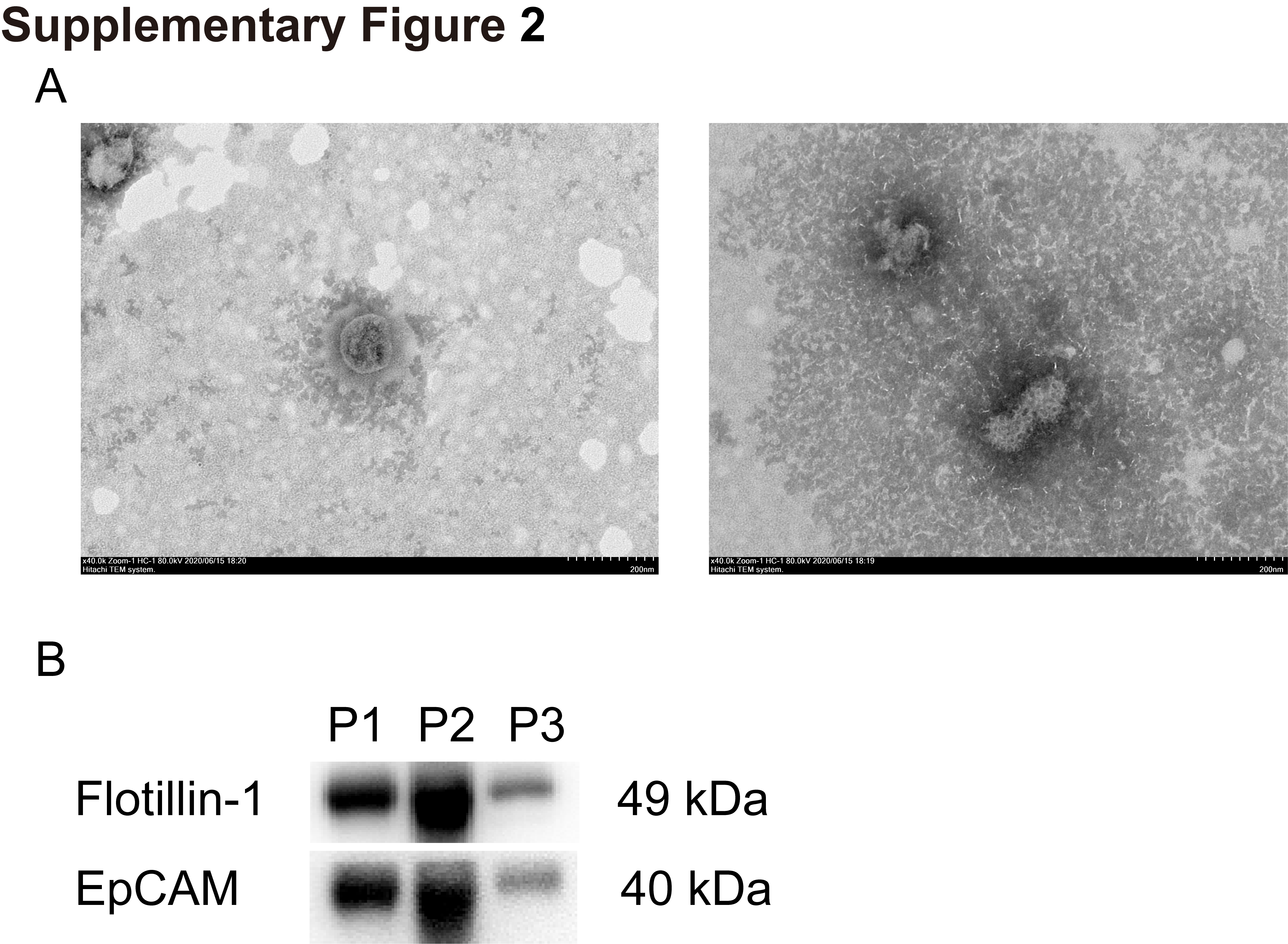

Supplement: Supplementary file 3 — Supplementary Figure S2. [file 41598_2023_38373_MOESM3_ESM.tif]

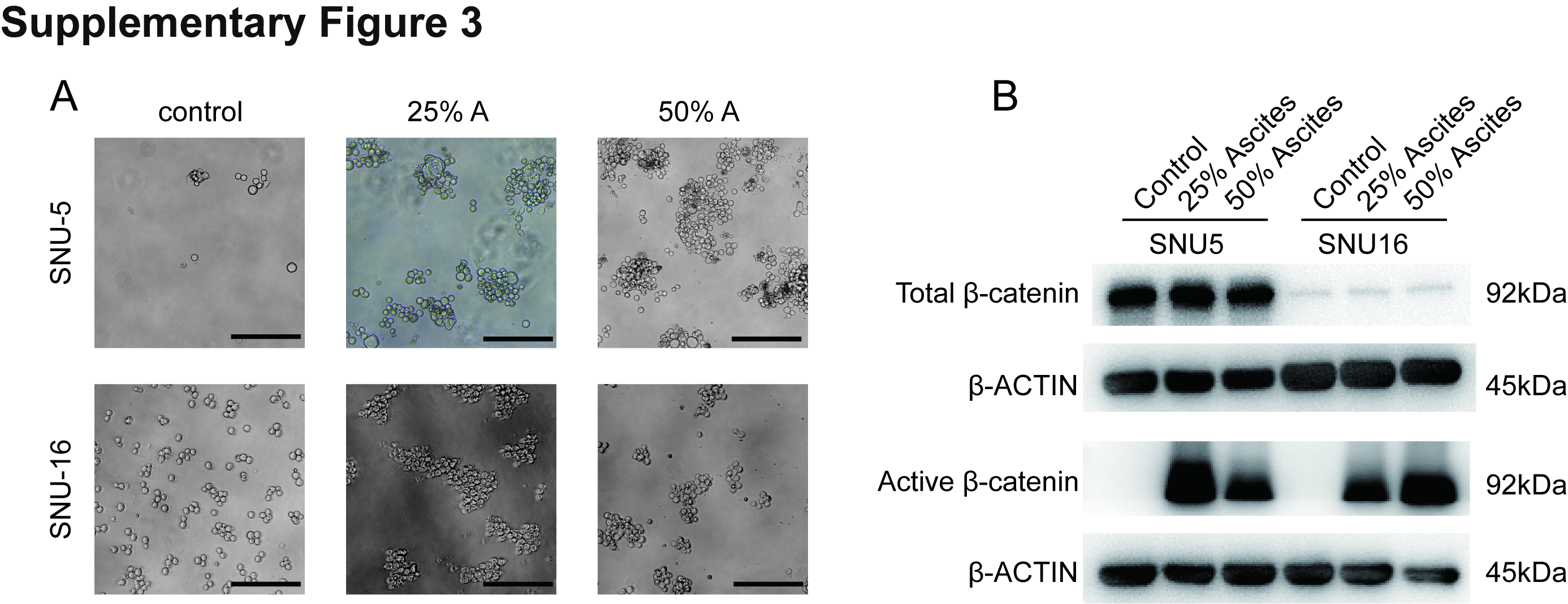

Supplement: Supplementary file 4 — Supplementary Figure S3. [file 41598_2023_38373_MOESM4_ESM.tif]

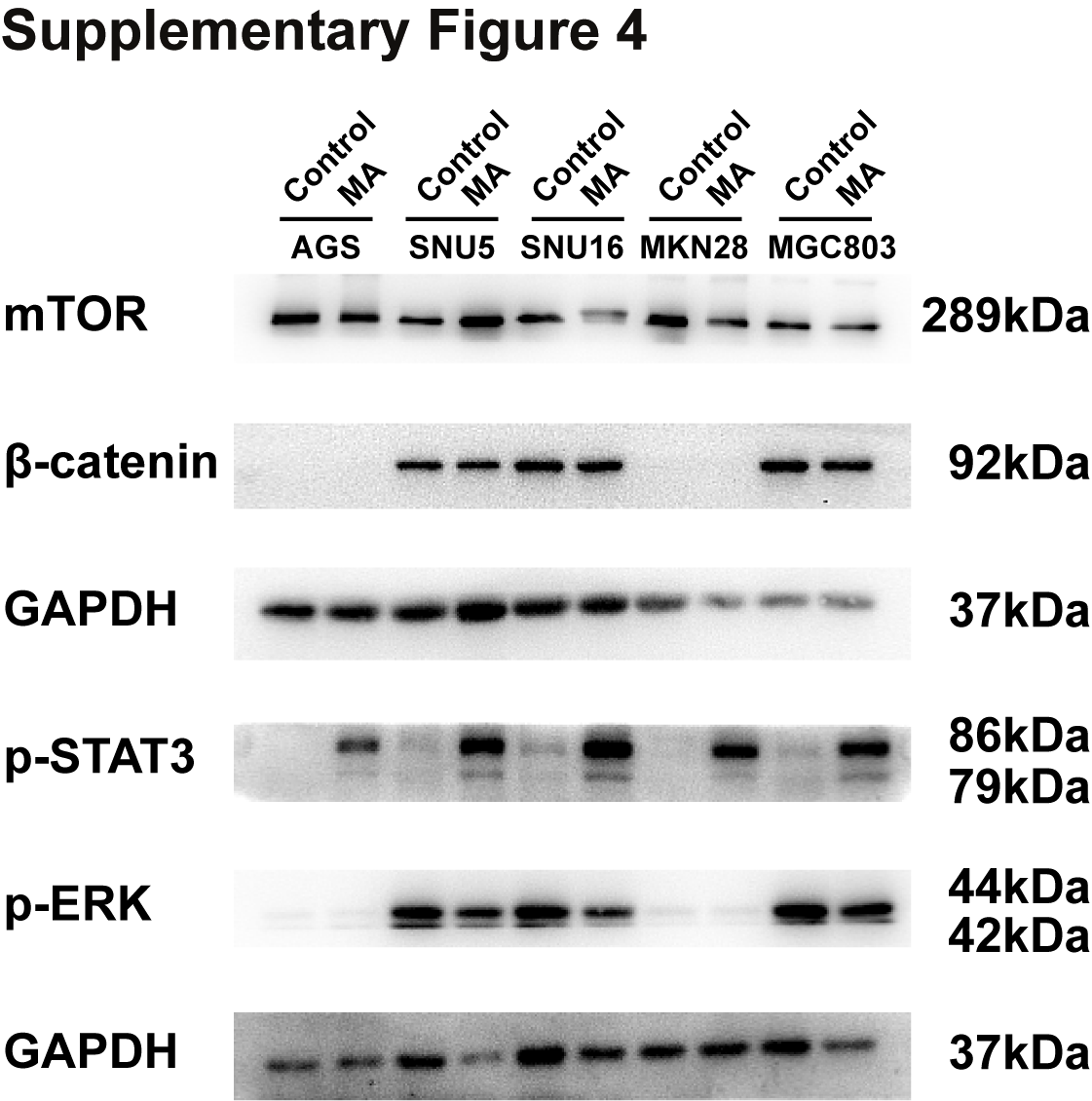

Supplement: Supplementary file 5 — Supplementary Figure S4. [file 41598_2023_38373_MOESM5_ESM.tif]

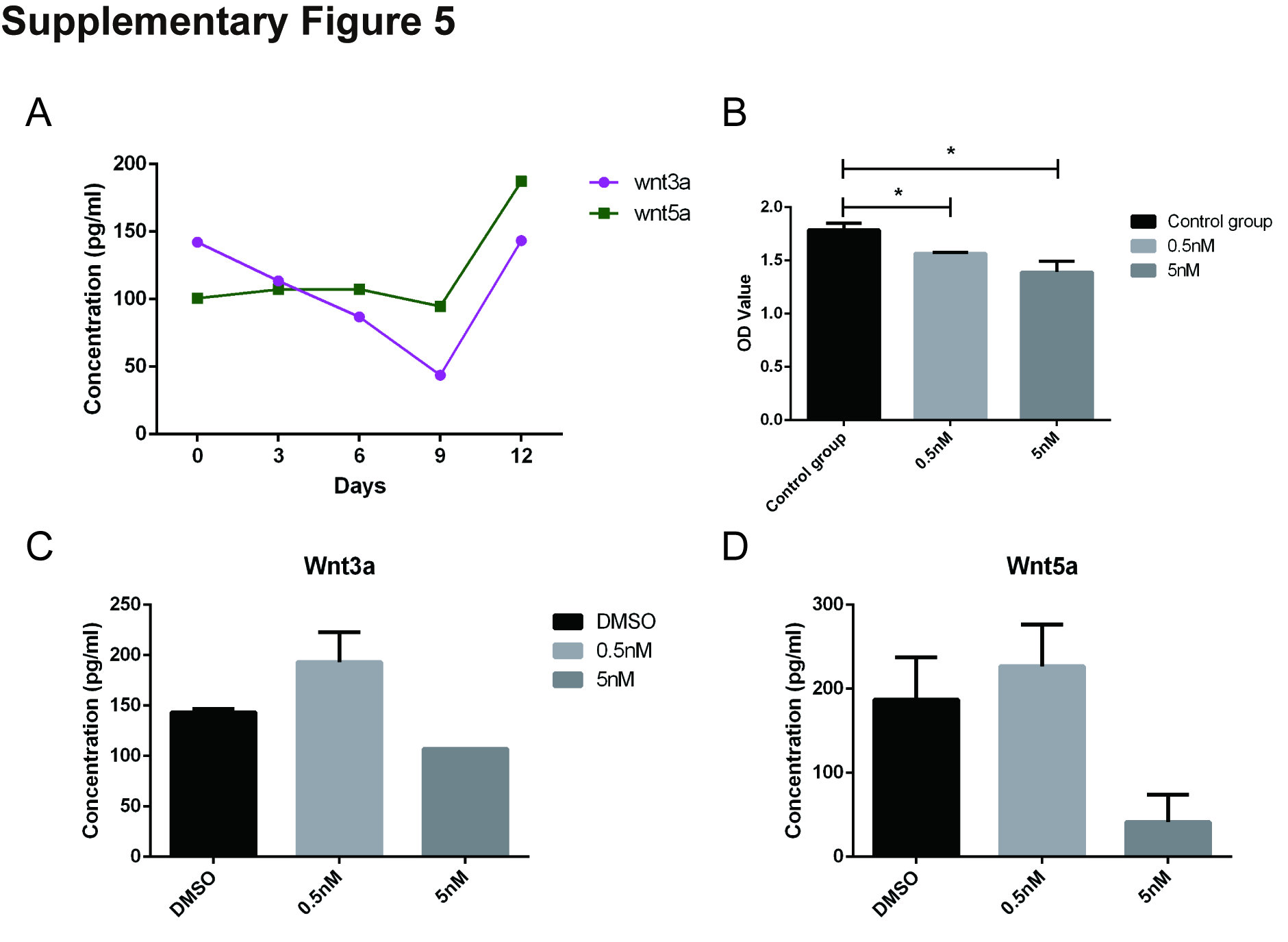

Supplement: Supplementary file 6 — Supplementary Figure S5. [file 41598_2023_38373_MOESM6_ESM.tif]

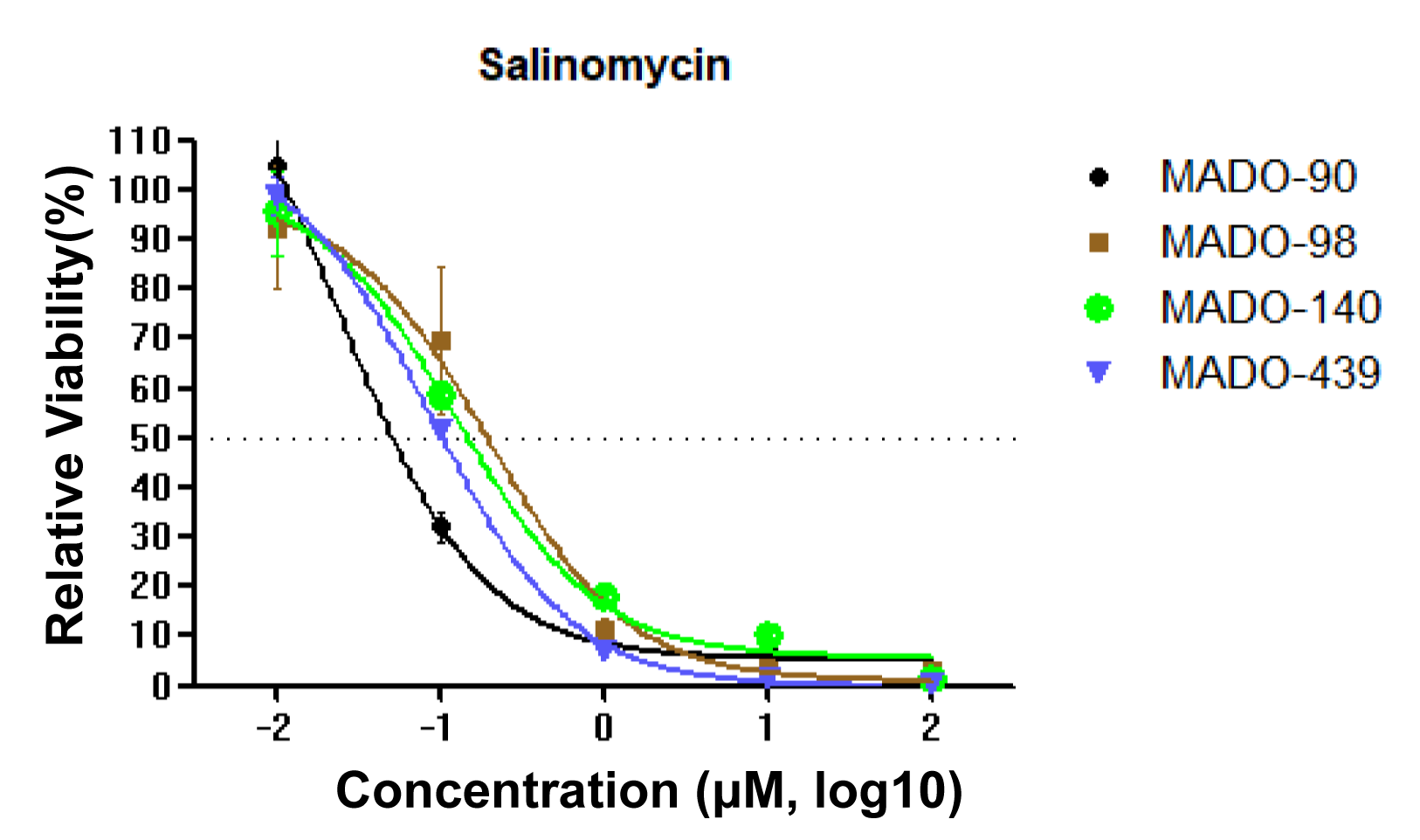

Supplement: Supplementary file 7 — Supplementary Figure S6. [file 41598_2023_38373_MOESM7_ESM.tif]
