# Supplementary material for: Liquid tumor microenvironment enhances WNT signaling pathway of peritoneal metastasis of gastric cancer
Source: Sci Rep. 2023 Jul 10;13:11125. doi: 10.1038/s41598-023-38373-6 (PMC10333202; doi:10.1038/s41598-023-38373-6)
Supplement: Supplementary file 8 — Supplementary Information. [file 41598_2023_38373_MOESM8_ESM.docx]

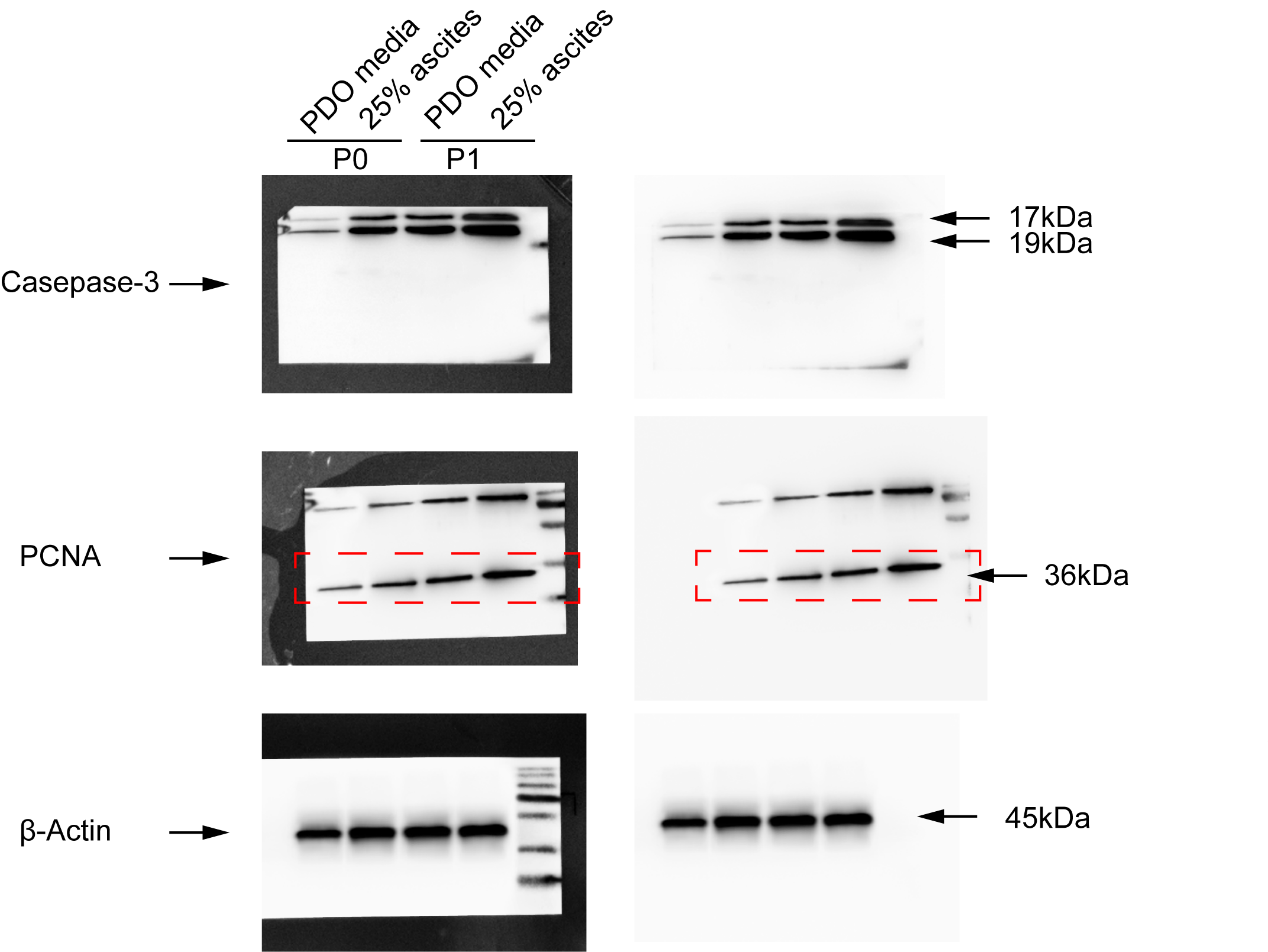


Original data of Figure 1G: Caspase-3 and PCNA expression levels in P0 and P1 MADOs treated with PDO media or PDO media containing 25% ascites supernatant were measured using western-blot assays. β-Actin was used as loading control to normalize the data.


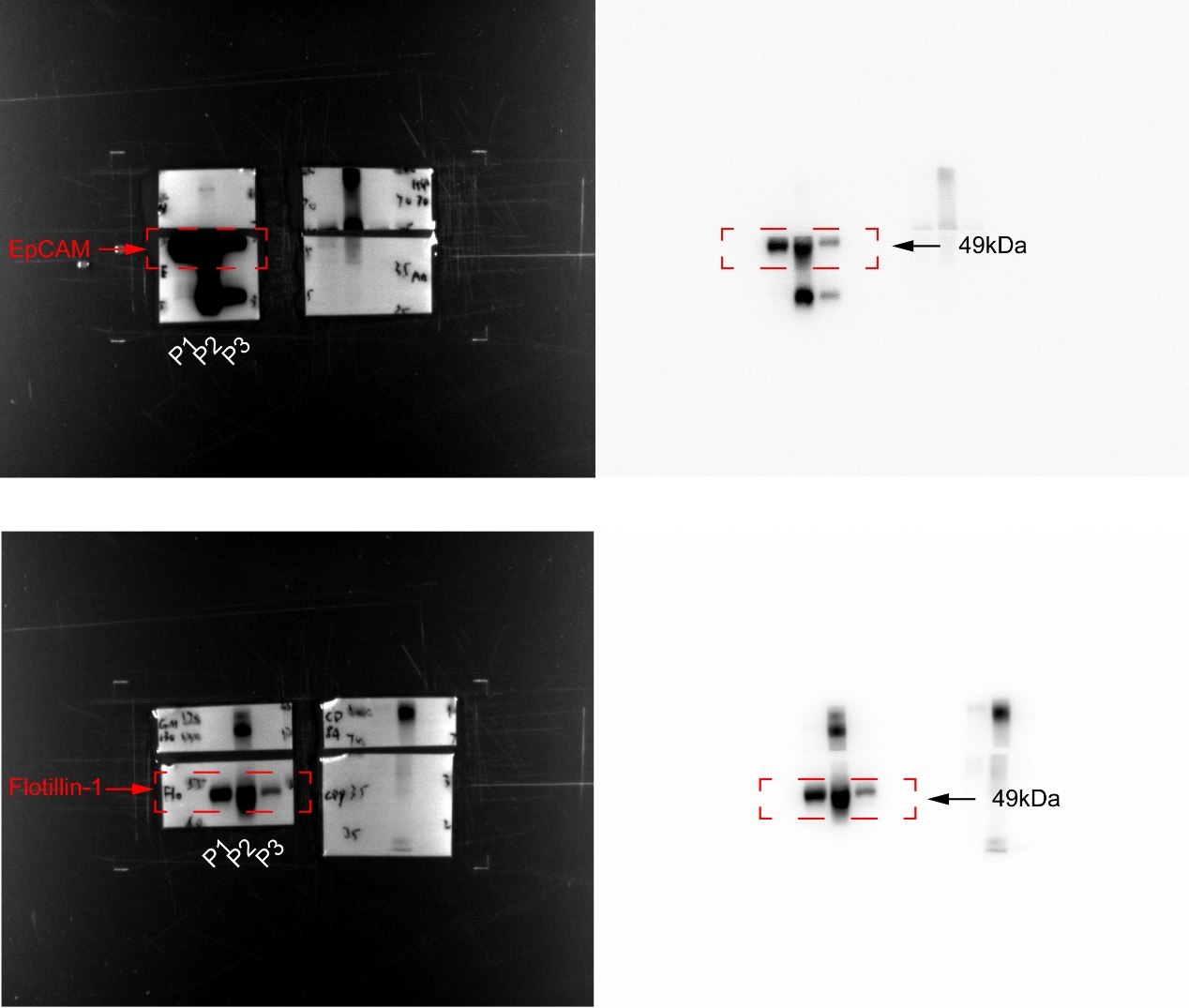


Original data of Supplementary Figure 2B: Western blot detection of proteins in ascites-derived exosomes using antibodies against exosomal markers: Flotillin-1, EpCAM. These exosomes derived from three gastric cancer patients (P1, P2, P3). Membranes were cut into several pieces (based on the molecular weight of proteins of interest) prior to hybridization with primary antibodies during blotting.


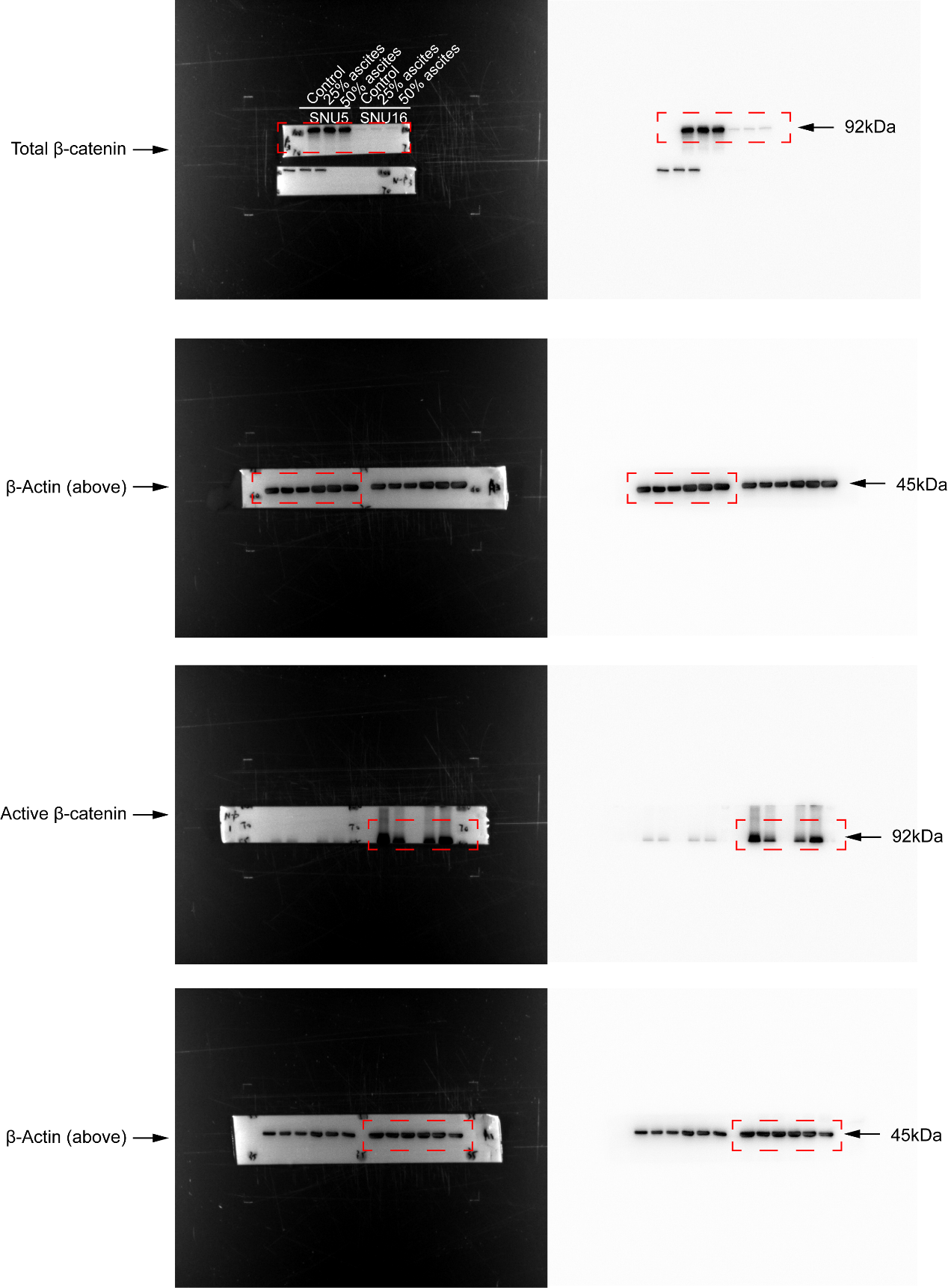


Original data of Supplementary Figure 3B: Western blotting analysis of total β-catenin and active β-catenin in SNU5 and SNU16 cell lines treated by 0% (control), 25% and 50% ascites. Membranes were cut into several pieces (based on the molecular weight of proteins of interest) prior to hybridization with primary antibodies during blotting. β-Actin was used as loading control to normalize the data.


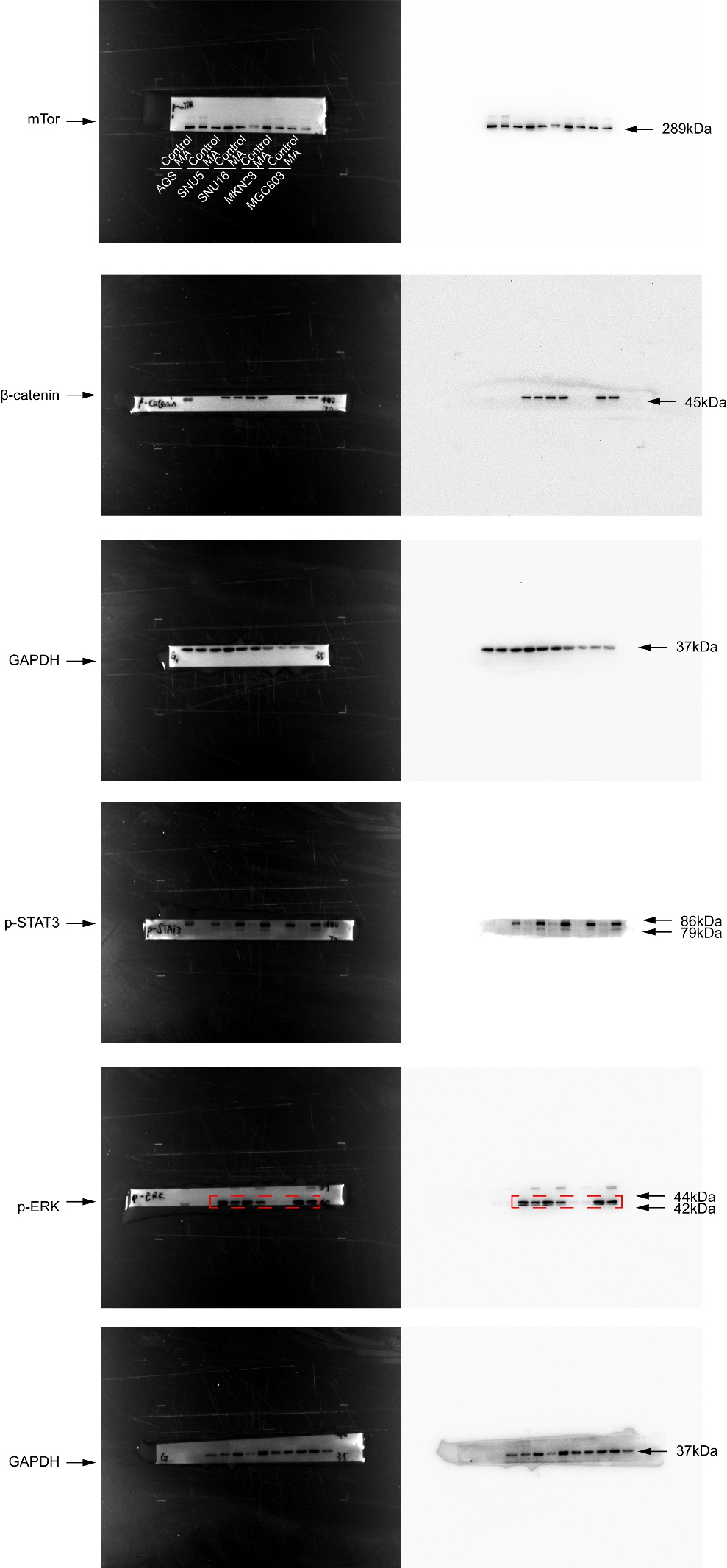


Original data of Supplementary Figure 4: Western blotting analysis of key proteins of several classical signaling pathway related to proliferation (mTor, β-catenin, p-STAT3, p-ERK) in several gastric cancer cell lines stimulated by ascites. Membranes were cut into several pieces (based on the molecular weight of proteins of interest) prior to hybridization with primary antibodies during blotting. GAPDH was used as loading control to normalize the data.
