# Supplementary material for: Liquid tumor microenvironment enhances WNT signaling pathway of peritoneal metastasis of gastric cancer
Source: Sci Rep. 2023 Jul 10;13:11125. doi: 10.1038/s41598-023-38373-6 (PMC10333202; doi:10.1038/s41598-023-38373-6)
Supplement: Supplementary file 9 — Supplementary Table S1. [file 41598_2023_38373_MOESM9_ESM.docx]

Table 1. MADO culture media

| **Additive** | **Supplier** | **Cat.NO.** | **Concentration** |
| --- | --- | --- | --- |
| wnt3a conditioned medium | home made |  | 50% |
| R-Spondin 1 | Sino Biological | 11083-HNAS | 500 ng·ml-1 |
| FGF 10 | Peprotech | 100-26 | 10 ng·ml-1 |
| EGF | Sino Biological | 10605-HNAE | 50 ng·ml-1 |
| Noggin | Sino Biological | 10267-HNAH | 100 ng·ml-1C |
| A83-01 | Tocris | 2939 | 2000 nM |
| Y-27632 | Tocris | 1254-bulk | 10 μM |
| Gastrin | Sigma | 39024-57-2 | 1 nM |
| B27 supplement | Gibco | 17504-44 | 1x |
| N-Acetylcysteine | Sigma | A9165-5g | 1 mM |
| Nicotinamide | Sigma | N0636 | 10 mM |
| GlutaMax 100x | Invitrogen | 12634-034 | 1x |
| Hepes | Invitrogen | 15630-056 | 10 mM |
| Penicillin/Streptomycin | Invitrogen | 15140-122 | 100 U·ml-1 / 100 mg·ml-1 |
| Primocin | Invitrogen | Ant-pm-1 | 50 mg·ml-1 |
| Advanced DMEM/F12 | Invitrogen | 12634-034 | 1x |
