# Supplementary material for: Liquid tumor microenvironment enhances WNT signaling pathway of peritoneal metastasis of gastric cancer
Source: Sci Rep. 2023 Jul 10;13:11125. doi: 10.1038/s41598-023-38373-6 (PMC10333202; doi:10.1038/s41598-023-38373-6)
Supplement: Supplementary file 10 — Supplementary Table S2. [file 41598_2023_38373_MOESM10_ESM.docx]

**Table 2 Concentrations of wnt3a/wnt5a in MADOs culture media**

| **Group(pg/ml)** | **Day0** | **Day3** | **Day6** | **Day9** |
| --- | --- | --- | --- | --- |
| **P0-PDO media** | 31.99±2.482 | 6.606±1.630 | 30.68±3.090 | 2.871±1.532 |
| **P0-25%Ascites** | 142.3±3.782 | 63.04±1.338 | 89.20±1.401 | 109.8±3.617 |
| **P value** | 0.0008 | 0.0007 | 0.0017 | 0.0007 |
| **P1-PDO media** | 31.99±2.482 | 40.43±1.907 | 30.23±1.234 | 29.39±6.154 |
| **P1-25%Ascites** | 142.3±3.782 | 133.8±3.740 | 102.7±2.147 | 92.18±2.816 |
| **P value** | 0.0008 | 0.0010 | 0.0006 | 0.0058 |

Wnt3a

| **Group(pg/ml)** | **Day0** | **Day3** | **Day6** | **Day9** |
| --- | --- | --- | --- | --- |
| **P0-PDO media** | 21.40±2.358 | 11.78±0.6380 | 14.92±3.801 | 7.585±0.5475 |
| **P0-25%Ascites** | 100.8±4.145 | 64.24±2.920 | 67.98±1.193 | 67.15±2.374 |
| **P value** | 0.0018 | 0.0016 | 0.0028 | 0.0008 |
| **P1-PDO media** | 21.40±2.358 | 14.15±2.717 | 7.051±1.864 | 12.72±1.964 |
| **P1-25%Ascites** | 100.8±4.145 | 97.40±4.773 | 73.55±1.842 | 84.71±3.882 |
| **P value** | 0.0018 | 0.0022 | 0.0008 | 0.0018 |

Wnt5a
